# Anti-TNF agents and potential effects on male fertility: are men being counseled?

**DOI:** 10.1186/s12894-020-00658-7

**Published:** 2020-07-27

**Authors:** Lauren Folgosa Cooley, James Wren, Mary Kate Keeter, Isaac Lam, Nelson Bennett, Robert E. Brannigan

**Affiliations:** Department of Urology, Northwestern University Feinberg School of Medicine, 676 N. St. Clair St. Arkes 23-015, Chicago, IL 60611 USA

**Keywords:** Infertility, Counseling, Anti-TNF, Sperm cryopreservation, Autoimmune

## Abstract

**Background:**

Adult men with autoimmune conditions are commonly prescribed anti-tumor necrosis factor (anti-TNF) agents; however, there is a paucity of quality evidence as to their effect on male fertility (e.g. semen parameters and sperm quality). Our objective was to determine if men with autoimmune conditions are being counseled regarding the unknown reproductive effects of anti-TNF agents prior to initiation of therapy.

**Methods:**

A retrospective analysis of 1010 male patients age 18–45 who were prescribed an anti-TNF agent were assessed for (1) receipt of counseling regarding potential reproductive effects; (2) screening for anatomic or laboratory abnormalities associated with infertility; (3) election for sperm cryopreservation.

**Results:**

Only 10.3% of men received counseling, and this was not associated with age (*p* = 0.77). Those who received counseling were significantly more likely to have a genitourinary exam performed, be assessed for presence of a varicocele, be asked about or endorse low libido or erectile dysfunction, have a testosterone, LH, FSH, or prolactin level checked, and have a semen analysis performed (all, *p* < 0.0001). Rates of sperm cryopreservation were low, but statistically higher in men who received counseling (5.77% (+) counseling, 1.10% (−) counseling) (*p* = 0.002).

**Conclusions:**

The limited current literature lacks a consensus regarding the short- and long-term male reproductive effects of anti-TNF therapy. Despite this lack of clarity, rates of pre-initiation counseling were low. Rates of sperm cryopreservation, while improved in the counseled group remained low, suggesting prescribing physicians may be unaware of this option for patients.

## Background

Tumor necrosis factor (TNF) blockade has revolutionized the treatment of autoimmune conditions such as inflammatory joint and bowel disease, helping to reduce disease-associated morbidity and mortality [1]. However, the impact of these agents on male fertility is unclear based on the limited current literature. While the role of TNF in male spermatogenesis is still under investigation, the basic science literature suggests TNF plays critical role in spermatogenesis and maintaining the blood-testis barrier [2–4]. TNF is produced by germ cells and acts as a paracrine cytokine, binding to both Sertoli and Leydig cells [2–4]. Suominen et al. demonstrated that TNF is important in regulating germ cell apoptosis by promoting germ cell survival, which is blocked by infliximab (a TNF antagonist) [5]. Furthermore, sperm production was found to be reduced by 54% in a TNF-related-to-apoptosis-inducing ligand (TRAIL) deficient mouse model in whom TNF signaling is impaired [6]. While TNF is integral to spermatogenesis, if unregulated, high levels of TNF can also be detrimental by leading to testicular inflammation [7]. Therefore, maintaining an adequate equilibrium is essential.

The effect of TNF blockade on human fertility is poorly understood. While two small studies have shown no deleterious effect of anti-TNF agents on semen quality, spermatogenesis, production of anti-sperm antibodies, and testosterone levels [8, 9], others have demonstrated increased risk for asthenospermia (decreased sperm motility) [10–12] and impaired sperm morphology [12, 13]. In the rheumatology literature, concern has been raised of cases where sperm counts were significantly reduced following initiation of an anti-TNF agent [14]. Despite the “very low level of evidence” for the effects of anti-TNF agents on male fertility, a review of drug effects on male fertility by Semet et al. states that sperm cryopreservation and discontinuation of treatment for fertility concerns is not necessary for males initiating anti-TNF therapy [15].

Given the discordant research regarding specific reproductive effects, our objective was to ascertain if men prescribed anti-TNF agents are being: (1) counseled regarding potential for adverse reproductive effects; (2) screened for anatomic or laboratory abnormalities associated with infertility; and (3) electing for sperm cryopreservation.

## Methods

We performed an IRB-approved (STU00205946), retrospective chart review of 1010 male patients between the ages of 18 and 45 from January 2006 to September 2017 seen at a single academic medical center. Additional inclusion criteria included having never been prescribed an anti-TNF agent prior to the study period and being prescribed only one anti-TNF agent during the study period. Men prescribed multiple anti-TNF agents during the study period were excluded from analysis. Anti-TNF agents prescribed in this study were adalimumab, infliximab, etanercept, golimumab, and certolizumab.

Counseling was defined as a documented discussion (for example, office visit note or documented telephone encounter) between the patient and prescribing provider that the prescribed agent may pose a risk to reproduction or fertility. The counseling must have occurred prior to starting treatment. To assess if men were screened for anatomic or laboratory abnormalities associated with infertility, we determined if men received (1) a genitourinary exam, (2) screening for varicocele, (3) diagnosis or assessment for low libido, (4) testosterone, FSH, LH, and/or prolactin level, (5) semen analysis (separate from cryopreservation), and (6) sperm cryopreservation. Genitourinary examination was confirmed by a documented assessment of the penis, meatus, and/or testes. Varicocele screening must have included a documentation of absence of varicocele or a grading score and laterality of assessed varicocele by palpation. Sperm cryopreservation included only men who elected cryopreservation. All six parameters may have occurred at the initial visit when the anti-TNF agent was prescribed or during their course of treatment (not necessarily by prescribing provider). Demographic information including marital status, race, and ethnicity were determined from the medical record. Patient diagnosis was defined as prescribing clinician diagnosis entered in the medical record at the time the anti-TNF agent was prescribed.

Each pharmaceutical approved for use in the United States by the Food and Drug Administration (FDA) has a corresponding highlights of prescribing information sheet, which includes information regarding known impact of a drug on fertility. Highlights of prescribing information sheets were assessed for statements and literature references regarding impact of each anti-TNF agent on male fertility (Table 2).

Data was analyzed using GraphPad Prism. Categorical variables and proportion of events between those that did or did not receive counseling were compared using chi-square test. Means with standard deviation were compared using Student’s *t*-test after determining normal distribution (Shapiro-Wilk test). A *p* value ≤0.05 was considered significant.

## Results

### Demographics

A total of 1010 men met inclusion criteria for this study. Demographic information for included patients is provided in Table 1. In summary, average age was 32.4 years and mean duration of treatment was 832 days (2.3 years). The cohort was predominantly white (67.62%) and non-Hispanic (76.44%). The majority of participants were single (non-married) (58.51%). Men in this study were most likely to be diagnosed with psoriasis or psoriatic arthritis (35.05%), inflammatory bowel disease (Crohn’s [21.10%] and ulcerative colitis [17.62%]), or ankylosing spondylitis (10.99%). The most common prescribers were dermatologists, gastroenterologists, and rheumatologists.

**Table 1:** Demographics of study patients

	Adalimumab	Etanercept	Infliximab	Golimumab	Certolizumab	Total
**Number of patients**	600	161	219	8	22	1010
**Age** (years)	32.8 ± 7.5	33.0 ± 6.9	30.9 ± 7.4	33.9 ± 7.5	31.8 ± 7.1	32.4 ± 7.5
**Duration of treatment** (days)	799 ± 700	958 ± 878	853 ± 850	794 ± 725	669 ± 466	832 ± 806
**Race**	**n %**	**n %**	**n %**	**n %**	**n %**	**n %**
American Indian	1 (0.17)	0 (0.00)	2 (0.91)	0 (0.00)	0 (0.00)	3 (0.30)
Asian	16 (2.67)	5 (3.11)	9 (4.10)	0 (0.00)	0 (0.00)	30 (2.97)
Black	29 (4.83)	4 (2.48)	27 (12.32)	0 (0.00)	0 (0.00)	60 (5.94)
Hispanic	1 (0.17)	1 (0.62)	2 (0.91)	0 (0.00)	0 (0.00)	4 (0.40)
Pacific Islander	1 (0.17)	0 (0.00)	0 (0.00)	0 (0.00)	0 (0.00)	1 (0.01)
White	404 (67.33)	99 (61.5)	153 (69.86)	8 (100)	19 (86.36)	683 (67.62)
Declined	78 (13.00)	36 (22.4)	9 (4.10)	0 (0.00)	0 (0.00)	123 (12.18)
Other	70 (11.67)	16 (9.94)	17 (7.76)	0 (0.00)	3 (13.64)	106 (10.50)
**Ethnicity**	**n %**	**n %**	**n %**	**n %**	**n %**	**n %**
Hispanic	29 (4.83)	10 (6.21)	13 (5.93)	0 (0.00)	0 (0.00)	52 (5.15)
Not Hispanic/Latino	465 (77.50)	103 (63.98)	177 (80.82)	8 (100)	19 (86.36)	772 (76.44)
Declined	106 (17.67)	48 (29.81)	29 (13.24)	0 (0.00)	3 (13.64)	186 (18.41)
**Marital Status**	**n %**	**n %**	**n %**	**n %**	**n %**	**n %**
Single	342 (57.00)	77 (47.83)	153 (69.86)	6 (75.00)	13 (59.09)	591 (58.51)
Married	205 (34.17)	71 (44.10)	64 (29.22)	2 (25.00)	9 (40.91)	351 (34.75)
Divorced	4 (0.67)	3 (1.86)	0 (0.00)	0 (0.00)	0 (0.00)	7 (0.69)
Other	49 (8.17)	10 (6.21)	2 (0.91)	0 (0.00)	0 (0.00)	61 (6.04)
**Diagnosis**	**n %**	**n %**	**n %**	**n %**	**n %**	**n %**
Ankylosing spondylitis	61 (10.16)	42 (26.09)	7 (3.19)	0 (0.00)	1 (4.55)	111 (10.99)
Behcet’s	2 (0.33)	0 (0.00)	1 (0.46)	0 (0.00)	0 (0.00)	3 (0.29)
Blau Syndrome	2 (0.33)	0 (0.00)	0 (0.00)	0 (0.00)	0 (0.00)	2 (0.20)
Crohn’s	107 (17.83)	0 (0.00)	90 (41.09)	3 (37.50)	13 (59.09)	213 (21.10)
Hidradenitis suppurativa	25 (4.17)	1 (0.62)	1 (0.46)	0 (0.00)	0 (0.00)	27 (2.67)
Psoriasis ± arthritis	256 (42.67)	83 (51.55)	11 (5.02)	2 (25.00)	2 (9.09)	354 (35.05)
Rheumatoid arthritis	24 (4.00)	20 (12.42)	18 (8.22)	0 (0.00)	1 (4.55)	63 (6.24)
Sarcoidosis	2 (0.33)	0 (0.00)	12 (5.48)	0 (0.00)	0 (0.00)	14 (1.39)
Seronegative arthritis	28 (4.67)	11 (6.83)	0 (0.00)	0 (0.00)	1 (4.55)	40 (3.96)
Sjogren’s	1 (0.17)	2 (1.24)	2 (0.91)	0 (0.00)	0 (0.00)	5 (0.49)
Ulcerative Colitis	92 (15.33)	2 (1.24)	77 (35.16)	3 (37.50)	4 (18.18)	178 (17.62)

All participants were male and prescribed a single anti-TNF agent (adalimumab, etanercept, infliximab, golimumab, or certolizumab). Duration of treatment is presented as average ± standard deviation. Race and ethnicity categories reflect the options available for data insertion in Epic Systems

### Evaluation of anti-TNF agent prescribing information to consumers and clinicians

FDA prescribing information sheets for anti-TNF agents assessed in this study were evaluated for discussion of impact of each medication on male fertility (Table 2). The prescribing information for the three most common prescribed agents in this study, adalimumab, infliximab and etanercept, stated the impact of these agents on fertility is unknown [16–18]. While animal studies have been performed to evaluate fertility following use of golimumab and certolizumab, there is no reference, study results, or study methods provided for critical review [19, 20].

**Table 2:** Impact of anti-TNF agents on fertility from FDA prescribing information

Anti-TNF agent (Generic / Brand name)	Prescribing information
Adalimumab / Humira©	“Long-term animal studies of HUMIRA have not been conducted to evaluate the carcinogenic potential or its effect on fertility” [16].
Infliximab / Remicade©	“It is not known whether infliximab can impair fertility in humans” [17].
Etanercept / Enbrel©	“Long-term animal studies have not been conducted to evaluate the carcinogenic potential of etanercept or its effect on fertility” [18].
Golimumab / Simponi©	“A fertility study conducted in mice using an analogous anti-mouse TNFα antibody showed no impairment to fertility” [19].
Certolizumab / Cimzia©	“The cTN3 PF (TNFα pegylated Fab fragment) had no effects on the fertility and general reproductive performance of male and female rats at intravenous doses up 100 mg/kg, administered twice weekly” [20].

FDA prescribing information for each anti-TNF agent listed was analyzed for discussion of impact of each agent on male fertility

### Men prescribed anti-TNF agents rarely receive counseling regarding potential impact on fertility

Prior to starting an anti-TNF agent for the first time, 10.3% of men received counseling regrading potential impact on fertility (Table 3). Men who received counseling were significantly more likely to have a genitourinary exam performed, be screened for a varicocele, be asked about concerns with libido or sexual function, have a testosterone, LH, FSH or prolactin level checked, have a semen analysis performed, and elect for sperm cryopreservation (Table 3). Men who received counseling were significantly more likely to elect for sperm cryopreservation, but rates of cryopreservation were low in both groups (5.77% (+) counseling, 1.10% (−) counseling). Age was not a statistically significant factor related to likelihood to receive counseling (*p* = 0.77).

**Table 3:** Men with pre-initiation counseling are more likely to undergo reproductive assessment and sperm cryopreservation

Counseling	Received		Did not receive		p
	104/1010	(10.3%)	906/1010	(89.7%)	< 0.0001
**Components of history and examination**					
GU Exam	83/104	(79.8%)	267/906	(29.4%)	< 0.0001
Varicocele	26/104	(25.0%)	17/906	(1.9%)	< 0.0001
Libido/Sexual Function	46/104	(44.2%)	108/906	(11.9%)	< 0.0001
**Laboratory Assessment**					
Serum Hormone Testing	29/104	(27.9%)	28/906	(3.1%)	< 0.0001
Semen Analysis	18/104	(17.3%)	26/906	(2.9%)	< 0.0001
**Sperm Cryopreservation**	6/104	(5.7%)	10/906	(1.1%)	0.002

104 men received counseling compared to 906 men who did not receive counseling. *p* < 0.05 considered significant

## Discussion

Anti -TNF agents are commonly prescribed to young adult men with autoimmune conditions on a long-term basis. While it is known on a molecular level that TNF is required for optimal spermatogenesis, the literature currently lacks large cohort, randomized, prospective evidence to adequately answer this question. Furthermore, according to the FDA highlights of prescribing information for each anti-TNF agent evaluated in this study, the majority stated the impact of the agent on fertility was unknown. Those that reported no risk did not provide literature or study reference to allow for evaluation of their data (Table 2). Despite this lack of quality of evidence, the recommendations found in the literature suggest sperm cryopreservation or discontinuation of anti-TNF therapy for male fertility concerns is not necessary [15]. This discordant information available to prescribers in the literature may help to explain why only 10.3% of over 1000 men prescribed an anti-TNF agent in our study cohort received counseling as to potential adverse effects on fertility. This suggests that patients and clinicians may be unaware of this knowledge gap or may not be documenting the fertility aspect of their discussion.

Furthermore, men prescribed anti-TNF agents are a unique population in that their primary disease (e.g. inflammatory bowel disease (IBD) or ankylosing spondylitis) can affect sexual function and desire, which could also impact their fertility. Men with IBD, for example, have been shown to have increased rates of hypogonadism, reduced sexual interest, difficulty achieving or maintaining erection, and higher rates of depression compared to age matched controls [21–24]. Additionally, men with ankylosing spondylitis suffer from debilitating arthritis, spinal stiffness, and loss of spinal mobility which has been associated with impaired erectile function, arousal, and sexual satisfaction [25]. Our study demonstrated that the majority of men were not asked about libido or sexual function, which is a common problem that can significantly impact quality of life. Furthermore, while hypogonadism has been associated with increased systemic inflammatory markers and risk of autoimmune conditions in men, restoration of eugonadism has not been demonstrated to our knowledge with control of underlying autoimmune disease [26]. For example, Cutolo et al. demonstrated that use of anti-TNF agents did not change systemic levels of sex androgens in men with rheumatoid arthritis [27].

We found that if men received counseling regarding potential for adverse reproductive effects when taking anti-TNF agents, they were significantly more likely to be screened for anatomic abnormalities (e.g. meatal stenosis, varicocele, small testis volume) and have laboratory tests associated with infertility (e.g. testosterone level, semen analysis) performed. While this is only an association, it suggests that discussion of fertility may prompt further examination and workup to screen for other factors that may affect fertility. Lastly, rates of sperm cryopreservation were significantly higher in men who received counseling but were very low overall (< 6%). This suggests that clinicians may not be aware of this option or how best to approach the topic with patients, which has been demonstrated in other patient populations, such as men diagnosed with cancer [28, 29].

This study is limited by its retrospective nature, reliance on documentation by providers, and being a single institution study. Our data suggests that the impact of anti-TNF agents on male fertility is largely unknown as reported by the FDA prescribing information for each agent. Furthermore, it suggests rates of counseling regarding this topic are low. Given our study is from a single center only, we cannot generalize our findings to education received by all men who are started on anti-TNF therapy. Additionally, information available to providers and practice patterns may have changed over the timeline of this study (2006–2017). Until further research delineates the effect of anti-TNF agents on male fertility, we urge clinicians to counsel patients about this lack of evidence and discuss the option of fertility preservation measures, out of an abundance of caution, prior to starting treatment.

## Conclusions

The impact of anti-TNF agents on human male fertility is unclear, with the basic science and clinical literature published to date lacking conclusive evidence on the short and long term reproductive effects of anti-TNF therapy. Despite this lack of clarity, our data suggests that men are not being counseled regarding the potential for adverse reproductive outcomes. Furthermore, rates of sperm cryopreservation and discussion of sexual history in this population were low. A large, prospective study assessing the impact of anti-TNF agents on semen parameters, sexual health, and overall fertility outcomes is needed in order to better identify impacts of therapy, so physicians can more effectively counsel patients.

## Data Availability

The datasets used and/or analysed during the current study are available from the corresponding author on reasonable request. FDA information for anti-TNF agents can be found at the following links: https://www.accessdata.fda.gov/drugsatfda_docs/label/2011/125057s0276lbl.pdf https://www.accessdata.fda.gov/drugsatfda_docs/label/2013/103772s5359lbl.pdf https://www.accessdata.fda.gov/drugsatfda_docs/label/2012/103795s5503lbl.pdf https://www.accessdata.fda.gov/drugsatfda_docs/label/2011/125289s0064lbl.pdf https://www.accessdata.fda.gov/drugsatfda_docs/label/2017/125160s270lbl.pdf

## Abbreviations

IBD: Inflammatory bowel disease
FDA: Food and Drug Administration
FSH: Follicle stimulating hormone
LH: Luteinizing hormone
TNF: Tumor necrosis factor
TRAIL: TNF-related-to-apoptosis-inducing ligand
